# Systematic assessment of advanced respiratory physiology: precision medicine entering real-life ICU?

**DOI:** 10.1186/s13054-017-1720-3

**Published:** 2017-06-16

**Authors:** Tommaso Mauri, Giacomo Grasselli, Antonio Pesenti

**Affiliations:** 1 Department of Pathophysiology and Transplantation, University of Milan, Milan, Italy; 2 Department of Anesthesia, Critical Care and Emergency, Fondazione IRCCS Ca’ Granda Ospedale Maggiore Policlinico, Via F. Sforza 35, 20122 Milan, Italy; 3 Department of Pathophysiology and Transplantation, University of Milan, Milan, Italy

**Keywords:** Precision medicine, Respiratory mechanics, Positive end-expiratory pressure, Driving pressure, Transpulmonary pressure

In this uprising era of precision medicine [1], clinical translation of physiological measurements supporting personalized treatments in the intensive care unit (ICU) is of extreme interest. To this end, respiratory mechanics measurements in patients with acute respiratory distress syndrome (ARDS) might become standard to titrate mechanical ventilation settings [2]. This concept was the driving hypothesis of an interesting article by Lu Chen and colleagues recently published in *Critical Care* [3]. The authors report implementation into the real-life of the medical-surgical and trauma-neurosurgical ICUs of the Toronto-based St. Michael’s Hospital of a 1-year quality improvement program aimed at measuring advanced respiratory mechanics at the bedside in patients with ARDS. Output was real-time creation of an analytic report with actual patient measures handed to the attending physician and start of a prospective registry for future studies. The program enrolled 62 patients in the first year, all with early ARDS, deeply sedated and often paralyzed, who were switched to protective volume-controlled ventilation with standard settings. Esophageal pressure measure was added to patients with moderate and severe ARDS [4]. Target physiological measurements included in the clinical report and registry were: total positive end-expiratory pressure (PEEP), peak pressure, plateau pressure, intrinsic PEEP, driving pressure, respiratory system compliance, resistance, end-expiratory transpulmonary pressure, end-inspiratory transpulmonary pressure, lung compliance, chest wall compliance, transpulmonary plateau pressure, oxygenation, and hemodynamic response to a 3–5 cmH_2_O PEEP change [5], and (de)recruitment obtained at clinical PEEP by an abrupt 10 cmH_2_O PEEP decrease [6]. In the present analysis, at first the authors retrospectively looked at whether making these measurements available to the attending physician induced any change in ventilation settings. This was true in 67% of cases with a switch from pressure to volume control and PEEP change as the most frequent adjustments. Secondly, authors assessed whether the changes in ventilation settings ameliorated physiological variables known to be associated with patients’ clinical outcome: oxygenation index improved and plateau and driving pressure decreased. Finally, authors investigated whether the changes in ventilation settings were consistent with the physiological report findings and described how the attending physician introduced PEEP changes consistent with the indications suggested by the physiological assessments.

The study by Chen and colleagues obviously has limitations: it is a retrospective observational analysis describing an association between measuring advanced respiratory mechanics, changes in ventilation settings, and improvement of respiratory physiology that does not allow any description of causal relationship between these entities; it was performed in a single academic center with experience in conducting physiologic studies and clinical trials on mechanical ventilation for many years [5], making generalizability of the results difficult; the respiratory mechanics test was performed only once, while lung condition of ARDS patients can evolve rapidly towards improvement (with the need for decreasing ventilation support) or further decline (with the need for implementing rescue treatments such as prone positioning); the respiratory test was performed only in passive patients, while an early switch to assisted ventilation is increasingly implemented in ARDS patients and, during assisted breathing, respiratory mechanics and transpulmonary pressure critically depend upon patient’s efforts which was not mentioned in the target measures of this study [7]; finally, the test was not conducted by the attending physician but rather by dedicated personnel such as respiratory research therapists and clinical fellows, which might limit the application of such a program in facilities lacking these personnel.

Nonetheless, the study by Chen and colleagues represents a successful effort to bring advanced respiratory physiology to the bedside, targeted to a personalized therapy rather than to an “average” population effect. Since the results of the 6 vs. 12 ml/kg predicted body weight (PBW) tidal volume study in 2000 [8], research in mechanical ventilation for ARDS patients has suffered for more than a decade with “negative” clinical trials with, for example, no direct evidence of superiority of higher versus lower PEEP levels [9]. On the other hand, clinical respiratory physiology has greatly progressed, with deeper understanding of the mechanisms underlying ventilator-induced lung injury such as cyclic overdistension induced by elevated tidal volume/end-expiratory lung volume ratio [10] and with increasing clinical application of specific measures such as the estimate of the pleural pressure by the esophageal balloon [4]. These advancements have already translated into “positive” pilot clinical trials, showing that personalised PEEP titration or use of extracorporeal support based on target values of transpulmonary plateau pressure yielded better results than traditional care [11, 12], and into large observational studies describing stronger association between driving airway or transpulmonary pressure and outcome of ARDS patients in comparison to more traditional variables such as tidal volume per kilogram of PBW or plateau pressure [13–15]. Targeting PEEP to the level that grants (some) alveolar recruitment [10] without inducing (excessive) cyclic overdistension of the nondependent lung [6] and setting tidal volume based on the pressure distending the respiratory system or (even better) the lung within a protective threshold [15] might be the new standard of care for mechanical ventilation settings. However, translation into real-life would be impossible without the systematic assessment of respiratory mechanics at the bedside, such as the one reported by Dr. Chen and colleagues.

In conclusion, we need more evidence that personalized settings of PEEP and tidal volume based on advanced respiratory mechanics improves outcome of ARDS patients. At the same time, we also need evidence that such measures can be integrated into the real-life ICU workflow and can be used to titrate ventilator settings by real-life ICU doctors. To this end, the article by Lu Chen and colleagues has the evident merit to bring advanced respiratory physiology out of the laboratory and into the real world, and it is a valuable example for ICU teams interested in improving the care and outcome of ARDS.

## Abbreviations

ARDS: Acute respiratory distress syndrome
ICU: Intensive care medicine
PBW: Predicted body weight
PEEP: Positive end-expiratory pressure
